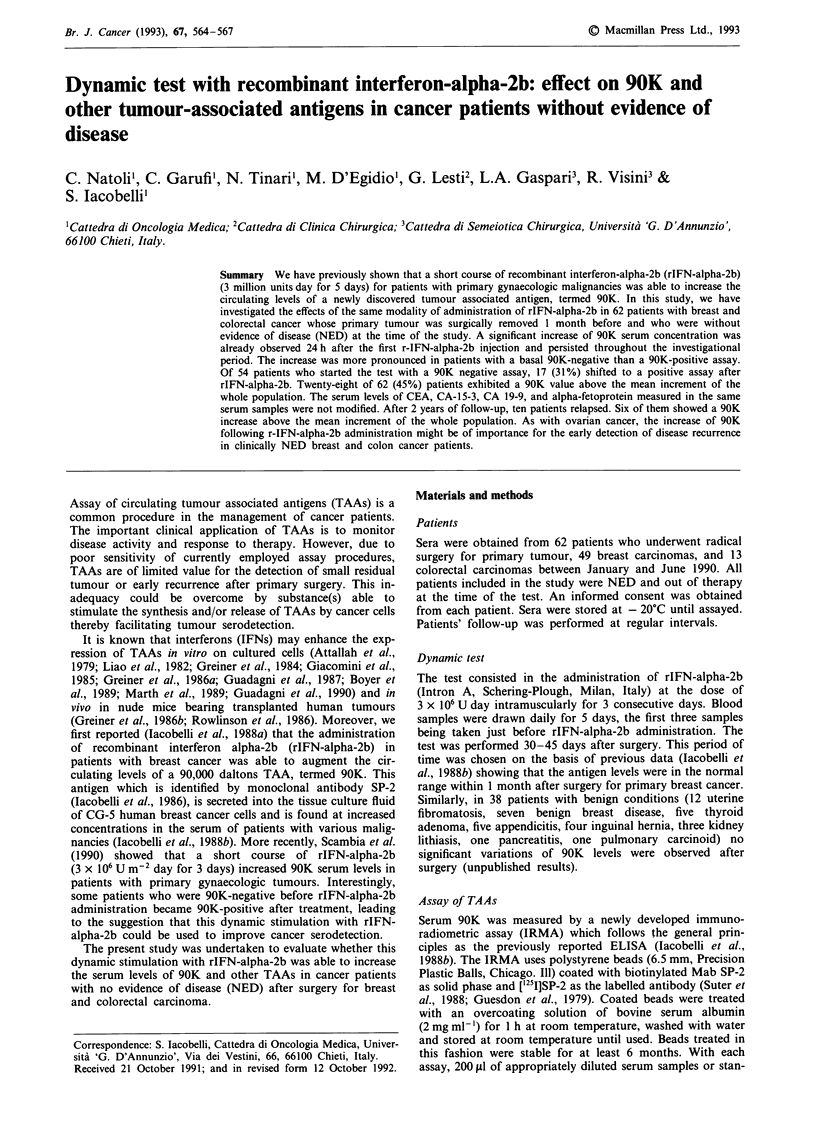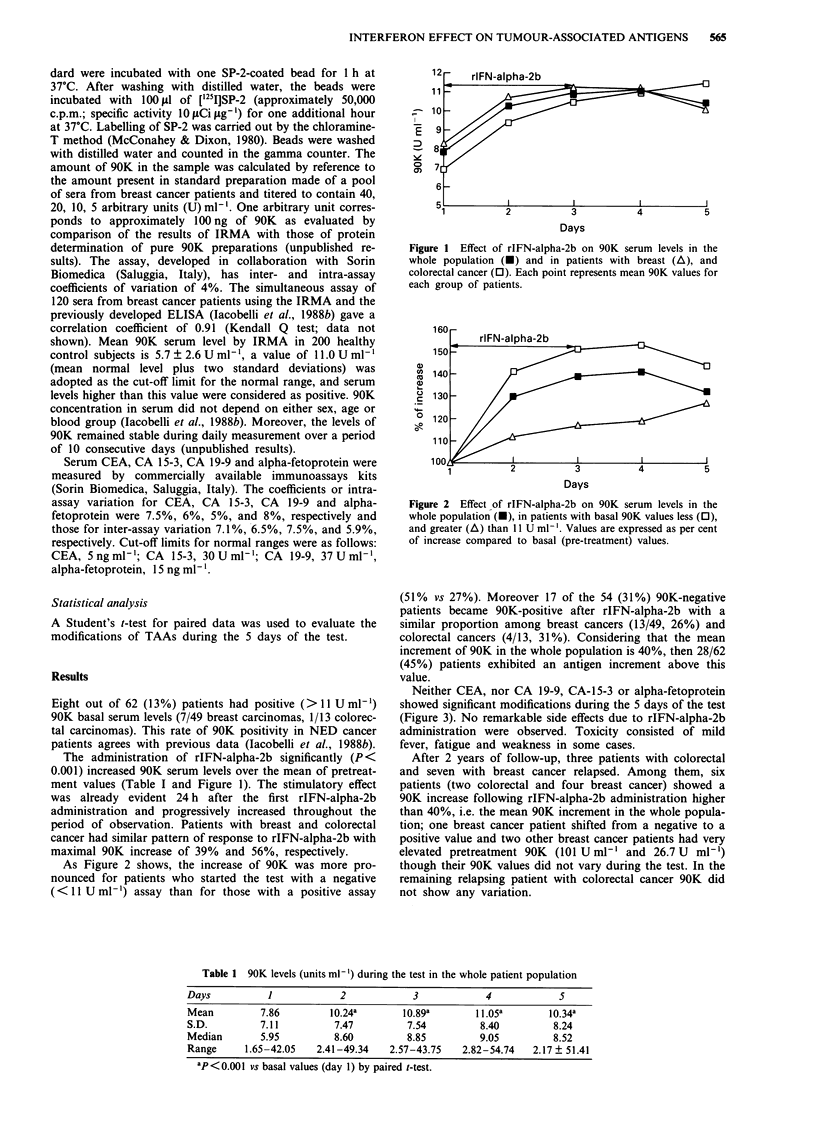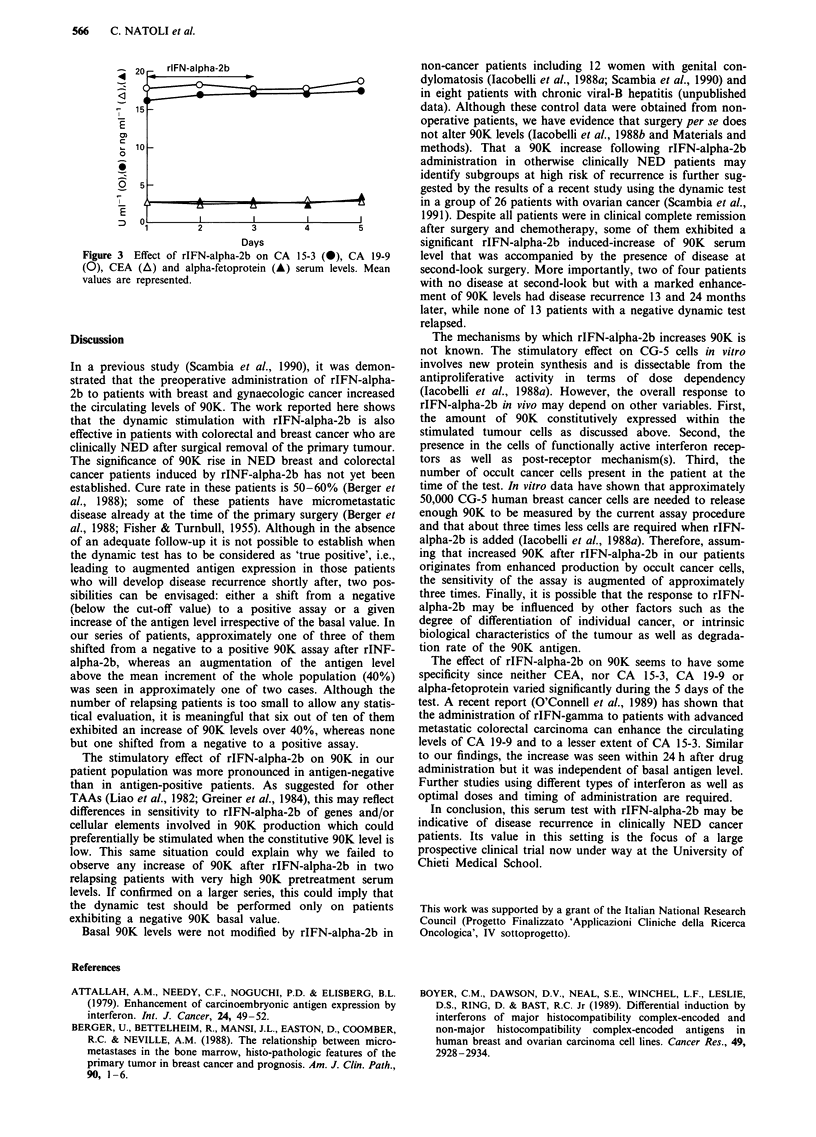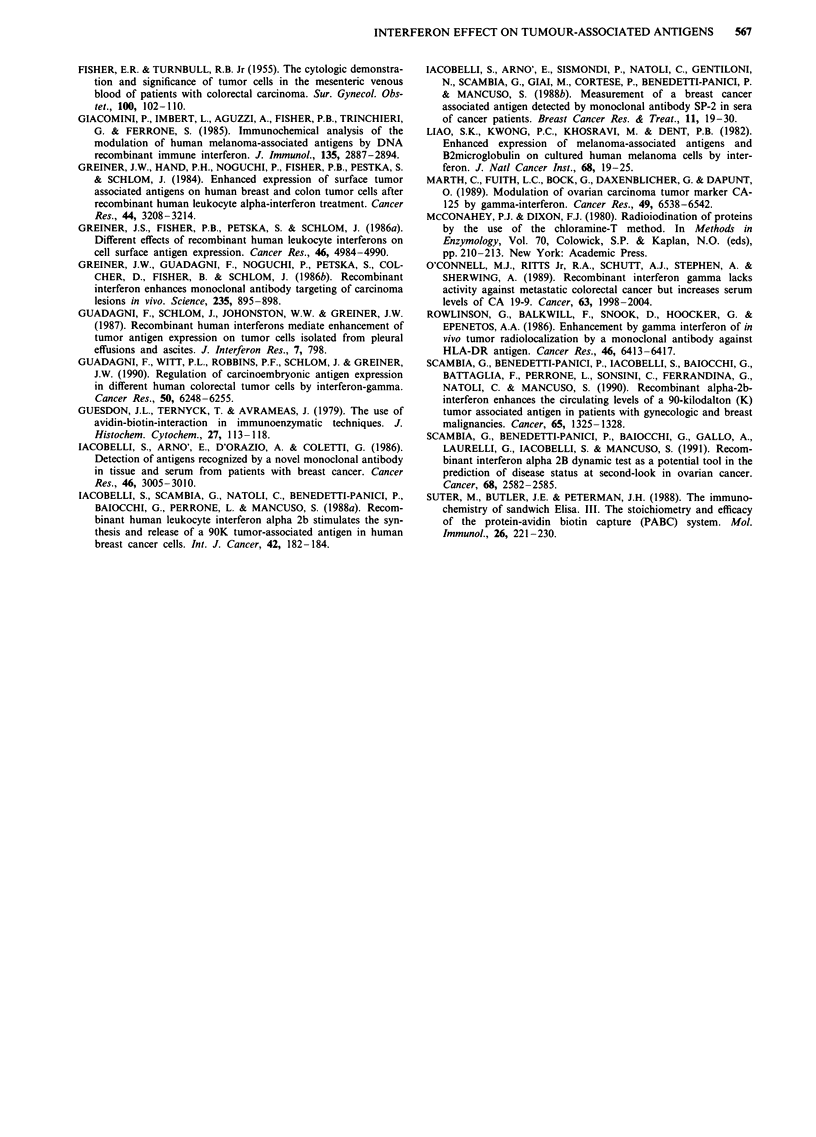# Dynamic test with recombinant interferon-alpha-2b: effect on 90K and other tumour-associated antigens in cancer patients without evidence of disease.

**DOI:** 10.1038/bjc.1993.103

**Published:** 1993-03

**Authors:** C. Natoli, C. Garufi, N. Tinari, M. D'Egidio, G. Lesti, L. A. Gaspari, R. Visini, S. Iacobelli

**Affiliations:** Cattedra di Oncologia Medica, Università G. D'Annunzio, Chieti, Italy.

## Abstract

We have previously shown that a short course of recombinant interferon-alpha-2b (rIFN-alpha-2b) (3 million units day for 5 days) for patients with primary gynaecologic malignancies was able to increase the circulating levels of a newly discovered tumour associated antigen, termed 90K. In this study, we have investigated the effects of the same modality of administration of rIFN-alpha-2b in 62 patients with breast and colorectal cancer whose primary tumour was surgically removed 1 month before and who were without evidence of disease (NED) at the time of the study. A significant increase of 90K serum concentration was already observed 24 h after the first r-IFN-alpha-2b injection and persisted throughout the investigational period. The increase was more pronounced in patients with a basal 90K-negative than a 90K-positive assay. Of 54 patients who started the test with a 90K negative assay, 17 (31%) shifted to a positive assay after rIFN-alpha-2b. Twenty-eight of 62 (45%) patients exhibited a 90K value above the mean increment of the whole population. The serum levels of CEA, CA-15-3, CA 19-9, and alpha-fetoprotein measured in the same serum samples were not modified. After 2 years of follow-up, ten patients relapsed. Six of them showed a 90K increase above the mean increment of the whole population. As with ovarian cancer, the increase of 90K following r-IFN-alpha-2b administration might be of importance for the early detection of disease recurrence in clinically NED breast and colon cancer patients.


Br. J. Cancer (1993), 67, 564–567 © Macmillan Press Ltd., 1993

### Dynamic test with recombinant interferon-alpha-2b: effect on 90K and other tumour-associated antigens in cancer patients without evidence of disease

C. Natoli[1], C. Garufi[1], N. Tinari[1], M. D'Egidio[1], G. Lesti[2], L.A. Gaspari[3], R. Visini[3] & S. Iacobelli[1]

[1]*Cattedra di Oncologia Medica;* [2]*Cattedra di Clinica Chirurgica;* [3]*Cattedra di Semeiotica Chirurgica, Università 'G. D'Annunzio', 66100 Chieti, Italy.*

**Summary** We have previously shown that a short course of recombinant interferon-alpha-2b (rIFN-alpha-2b) (3 million units day for 5 days) for patients with primary gynaecologic malignancies was able to increase the circulating levels of a newly discovered tumour associated antigen, termed 90K. In this study, we have investigated the effects of the same modality of administration of rIFN-alpha-2b in 62 patients with breast and colorectal cancer whose primary tumour was surgically removed 1 month before and who were without evidence of disease (NED) at the time of the study. A significant increase of 90K serum concentration was already observed 24 h after the first r-IFN-alpha-2b injection and persisted throughout the investigational period. The increase was more pronounced in patients with a basal 90K-negative than a 90K-positive assay. Of 54 patients who started the test with a 90K negative assay, 17 (31%) shifted to a positive assay after rIFN-alpha-2b. Twenty-eight of 62 (45%) patients exhibited a 90K value above the mean increment of the whole population. The serum levels of CEA, CA-15-3, CA 19-9, and alpha-fetoprotein measured in the same serum samples were not modified. After 2 years of follow-up, ten patients relapsed. Six of them showed a 90K increase above the mean increment of the whole population. As with ovarian cancer, the increase of 90K following r-IFN-alpha-2b administration might be of importance for the early detection of disease recurrence in clinically NED breast and colon cancer patients.

Assay of circulating tumour associated antigens (TAAs) is a common procedure in the management of cancer patients. The important clinical application of TAAs is to monitor disease activity and response to therapy. However, due to poor sensitivity of currently employed assay procedures, TAAs are of limited value for the detection of small residual tumour or early recurrence after primary surgery. This inadequacy could be overcome by substance(s) able to stimulate the synthesis and/or release of TAAs by cancer cells thereby facilitating tumour serodetection.

It is known that interferons (IFNs) may enhance the expression of TAAs *in vitro* on cultured cells (Attallah *et al.*, 1979; Liao *et al.*, 1982; Greiner *et al.*, 1984; Giacomini *et al.*, 1985; Greiner *et al.*, 1986*a*; Guadagni *et al.*, 1987; Boyer *et al.*, 1989; Marth *et al.*, 1989; Guadagni *et al.*, 1990) and *in vivo* in nude mice bearing transplanted human tumours (Greiner *et al.*, 1986*b*; Rowlinson *et al.*, 1986). Moreover, we first reported (Iacobelli *et al.*, 1988*a*) that the administration of recombinant interferon alpha-2b (rIFN-alpha-2b) in patients with breast cancer was able to augment the circulating levels of a 90,000 daltons TAA, termed 90K. This antigen which is identified by monoclonal antibody SP-2 (Iacobelli *et al.*, 1986), is secreted into the tissue culture fluid of CG-5 human breast cancer cells and is found at increased concentrations in the serum of patients with various malignancies (Iacobelli *et al.*, 1988*b*). More recently, Scambia *et al.* (1990) showed that a short course of rIFN-alpha-2b ($3 \times 10^6$ U $m^{-2}$ day for 3 days) increased 90K serum levels in patients with primary gynaecologic tumours. Interestingly, some patients who were 90K-negative before rIFN-alpha-2b administration became 90K-positive after treatment, leading to the suggestion that this dynamic stimulation with rIFN-alpha-2b could be used to improve cancer serodetection.

The present study was undertaken to evaluate whether this dynamic stimulation with rIFN-alpha-2b was able to increase the serum levels of 90K and other TAAs in cancer patients with no evidence of disease (NED) after surgery for breast and colorectal carcinoma.

Correspondence: S. Iacobelli, Cattedra di Oncologia Medica, Università 'G. D'Annunzio', Via dei Vestini, 66, 66100 Chieti, Italy.
Received 21 October 1991; and in revised form 12 October 1992.

### Materials and methods

### *Patients*

Sera were obtained from 62 patients who underwent radical surgery for primary tumour, 49 breast carcinomas, and 13 colorectal carcinomas between January and June 1990. All patients included in the study were NED and out of therapy at the time of the test. An informed consent was obtained from each patient. Sera were stored at – 20°C until assayed. Patients' follow-up was performed at regular intervals.

### *Dynamic test*

The test consisted in the administration of rIFN-alpha-2b (Intron A, Schering-Plough, Milan, Italy) at the dose of $3 \times 10^6$ U day intramuscularly for 3 consecutive days. Blood samples were drawn daily for 5 days, the first three samples being taken just before rIFN-alpha-2b administration. The test was performed 30–45 days after surgery. This period of time was chosen on the basis of previous data (Iacobelli *et al.*, 1988*b*) showing that the antigen levels were in the normal range within 1 month after surgery for primary breast cancer. Similarly, in 38 patients with benign conditions (12 uterine fibromatosis, seven benign breast disease, five thyroid adenoma, five appendicitis, four inguinal hernia, three kidney lithiasis, one pancreatitis, one pulmonary carcinoid) no significant variations of 90K levels were observed after surgery (unpublished results).

### *Assay of TAAs*

Serum 90K was measured by a newly developed immunoradiometric assay (IRMA) which follows the general principles as the previously reported ELISA (Iacobelli *et al.*, 1988*b*). The IRMA uses polystyrene beads (6.5 mm, Precision Plastic Balls, Chicago. Ill) coated with biotinylated Mab SP-2 as solid phase and [$^{125}$I]SP-2 as the labelled antibody (Suter *et al.*, 1988; Guesdon *et al.*, 1979). Coated beads were treated with an overcoating solution of bovine serum albumin (2 mg $ml^{-1}$) for 1 h at room temperature, washed with water and stored at room temperature until used. Beads treated in this fashion were stable for at least 6 months. With each assay, 200 $\mu$l of appropriately diluted serum samples or standard were incubated with one SP-2-coated bead for 1 h at 37°C. After washing with distilled water, the beads were incubated with 100 µl of [$^{125}$I]SP-2 (approximately 50,000 c.p.m.; specific activity 10 µCi µg$^{-1}$) for one additional hour at 37°C. Labelling of SP-2 was carried out by the chloramine-T method (McConahey & Dixon, 1980). Beads were washed with distilled water and counted in the gamma counter. The amount of 90K in the sample was calculated by reference to the amount present in standard preparation made of a pool of sera from breast cancer patients and titered to contain 40, 20, 10, 5 arbitrary units (U) ml$^{-1}$. One arbitrary unit corresponds to approximately 100 ng of 90K as evaluated by comparison of the results of IRMA with those of protein determination of pure 90K preparations (unpublished results). The assay, developed in collaboration with Sorin Biomedica (Saluggia, Italy), has inter- and intra-assay coefficients of variation of 4%. The simultaneous assay of 120 sera from breast cancer patients using the IRMA and the previously developed ELISA (Iacobelli *et al.*, 1988*b*) gave a correlation coefficient of 0.91 (Kendall Q test; data not shown). Mean 90K serum level by IRMA in 200 healthy control subjects is 5.7 ± 2.6 U ml$^{-1}$, a value of 11.0 U ml$^{-1}$ (mean normal level plus two standard deviations) was adopted as the cut-off limit for the normal range, and serum levels higher than this value were considered as positive. 90K concentration in serum did not depend on either sex, age or blood group (Iacobelli *et al.*, 1988*b*). Moreover, the levels of 90K remained stable during daily measurement over a period of 10 consecutive days (unpublished results).

Serum CEA, CA 15-3, CA 19-9 and alpha-fetoprotein were measured by commercially available immunoassays kits (Sorin Biomedica, Saluggia, Italy). The coefficients or intra-assay variation for CEA, CA 15-3, CA 19-9 and alpha-fetoprotein were 7.5%, 6%, 5%, and 8%, respectively and those for inter-assay variation 7.1%, 6.5%, 7.5%, and 5.9%, respectively. Cut-off limits for normal ranges were as follows: CEA, 5 ng ml$^{-1}$; CA 15-3, 30 U ml$^{-1}$; CA 19-9, 37 U ml$^{-1}$, alpha-fetoprotein, 15 ng ml$^{-1}$.

*Statistical analysis*

A Student's *t*-test for paired data was used to evaluate the modifications of TAAs during the 5 days of the test.

### Results

Eight out of 62 (13%) patients had positive (>11 U ml$^{-1}$) 90K basal serum levels (7/49 breast carcinomas, 1/13 colorectal carcinomas). This rate of 90K positivity in NED cancer patients agrees with previous data (Iacobelli *et al.*, 1988*b*).

The administration of rIFN-alpha-2b significantly ($P <$ 0.001) increased 90K serum levels over the mean of pretreatment values (Table I and Figure 1). The stimulatory effect was already evident 24 h after the first rIFN-alpha-2b administration and progressively increased throughout the period of observation. Patients with breast and colorectal cancer had similar pattern of response to rIFN-alpha-2b with maximal 90K increase of 39% and 56%, respectively.

As Figure 2 shows, the increase of 90K was more pronounced for patients who started the test with a negative (<11 U ml$^{-1}$) assay than for those with a positive assay

**Figure 1** Effect of rIFN-alpha-2b on 90K serum levels in the whole population (■) and in patients with breast (△), and colorectal cancer (□). Each point represents mean 90K values for each group of patients.

**Figure 2** Effect of rIFN-alpha-2b on 90K serum levels in the whole population (■), in patients with basal 90K values less (□), and greater (△) than 11 U ml$^{-1}$. Values are expressed as per cent of increase compared to basal (pre-treatment) values.

(51% *vs* 27%). Moreover 17 of the 54 (31%) 90K-negative patients became 90K-positive after rIFN-alpha-2b with a similar proportion among breast cancers (13/49, 26%) and colorectal cancers (4/13, 31%). Considering that the mean increment of 90K in the whole population is 40%, then 28/62 (45%) patients exhibited an antigen increment above this value.

Neither CEA, nor CA 19-9, CA-15-3 or alpha-fetoprotein showed significant modifications during the 5 days of the test (Figure 3). No remarkable side effects due to rIFN-alpha-2b administration were observed. Toxicity consisted of mild fever, fatigue and weakness in some cases.

After 2 years of follow-up, three patients with colorectal and seven with breast cancer relapsed. Among them, six patients (two colorectal and four breast cancer) showed a 90K increase following rIFN-alpha-2b administration higher than 40%, i.e. the mean 90K increment in the whole population; one breast cancer patient shifted from a negative to a positive value and two other breast cancer patients had very elevated pretreatment 90K (101 U ml$^{-1}$ and 26.7 U ml$^{-1}$) though their 90K values did not vary during the test. In the remaining relapsing patient with colorectal cancer 90K did not show any variation.

**Table 1** 90K levels (units ml$^{-1}$) during the test in the whole patient population

| *Days* | *1* | *2* | *3* | *4* | *5* |
|---|---|---|---|---|---|
| Mean | 7.86 | 10.24$^a$ | 10.89$^a$ | 11.05$^a$ | 10.34$^a$ |
| S.D. | 7.11 | 7.47 | 7.54 | 8.40 | 8.24 |
| Median | 5.95 | 8.60 | 8.85 | 9.05 | 8.52 |
| Range | 1.65–42.05 | 2.41–49.34 | 2.57–43.75 | 2.82–54.74 | 2.17 ± 51.41 |

$^a$$P <$0.001 *vs* basal values (day 1) by paired *t*-test.

**Figure 3** Effect of rIFN-alpha-2b on CA 15-3 (●), CA 19-9 (O), CEA (Δ) and alpha-fetoprotein (▲) serum levels. Mean values are represented.

### Discussion

In a previous study (Scambia *et al.*, 1990), it was demonstrated that the preoperative administration of rIFN-alpha-2b to patients with breast and gynaecologic cancer increased the circulating levels of 90K. The work reported here shows that the dynamic stimulation with rIFN-alpha-2b is also effective in patients with colorectal and breast cancer who are clinically NED after surgical removal of the primary tumour. The significance of 90K rise in NED breast and colorectal cancer patients induced by rINF-alpha-2b has not yet been established. Cure rate in these patients is 50–60% (Berger *et al.*, 1988); some of these patients have micrometastatic disease already at the time of the primary surgery (Berger *et al.*, 1988; Fisher & Turnbull, 1955). Although in the absence of an adequate follow-up it is not possible to establish when the dynamic test has to be considered as 'true positive', i.e., leading to augmented antigen expression in those patients who will develop disease recurrence shortly after, two possibilities can be envisaged: either a shift from a negative (below the cut-off value) to a positive assay or a given increase of the antigen level irrespective of the basal value. In our series of patients, approximately one of three of them shifted from a negative to a positive 90K assay after rINF-alpha-2b, whereas an augmentation of the antigen level above the mean increment of the whole population (40%) was seen in approximately one of two cases. Although the number of relapsing patients is too small to allow any statistical evaluation, it is meaningful that six out of ten of them exhibited an increase of 90K levels over 40%, whereas none but one shifted from a negative to a positive assay.

The stimulatory effect of rIFN-alpha-2b on 90K in our patient population was more pronounced in antigen-negative than in antigen-positive patients. As suggested for other TAAs (Liao *et al.*, 1982; Greiner *et al.*, 1984), this may reflect differences in sensitivity to rIFN-alpha-2b of genes and/or cellular elements involved in 90K production which could preferentially be stimulated when the constitutive 90K level is low. This same situation could explain why we failed to observe any increase of 90K after rIFN-alpha-2b in two relapsing patients with very high 90K pretreatment serum levels. If confirmed on a larger series, this could imply that the dynamic test should be performed only on patients exhibiting a negative 90K basal value.

Basal 90K levels were not modified by rIFN-alpha-2b in non-cancer patients including 12 women with genital condylomatosis (Iacobelli *et al.*, 1988*a*; Scambia *et al.*, 1990) and in eight patients with chronic viral-B hepatitis (unpublished data). Although these control data were obtained from non-operative patients, we have evidence that surgery *per se* does not alter 90K levels (Iacobelli *et al.*, 1988*b* and Materials and methods). That a 90K increase following rIFN-alpha-2b administration in otherwise clinically NED patients may identify subgroups at high risk of recurrence is further suggested by the results of a recent study using the dynamic test in a group of 26 patients with ovarian cancer (Scambia *et al.*, 1991). Despite all patients were in clinical complete remission after surgery and chemotherapy, some of them exhibited a significant rIFN-alpha-2b induced-increase of 90K serum level that was accompanied by the presence of disease at second-look surgery. More importantly, two of four patients with no disease at second-look but with a marked enhancement of 90K levels had disease recurrence 13 and 24 months later, while none of 13 patients with a negative dynamic test relapsed.

The mechanisms by which rIFN-alpha-2b increases 90K is not known. The stimulatory effect on CG-5 cells *in vitro* involves new protein synthesis and is dissectable from the antiproliferative activity in terms of dose dependency (Iacobelli *et al.*, 1988*a*). However, the overall response to rIFN-alpha-2b *in vivo* may depend on other variables. First, the amount of 90K constitutively expressed within the stimulated tumour cells as discussed above. Second, the presence in the cells of functionally active interferon receptors as well as post-receptor mechanism(s). Third, the number of occult cancer cells present in the patient at the time of the test. *In vitro* data have shown that approximately 50,000 CG-5 human breast cancer cells are needed to release enough 90K to be measured by the current assay procedure and that about three times less cells are required when rIFN-alpha-2b is added (Iacobelli *et al.*, 1988*a*). Therefore, assuming that increased 90K after rIFN-alpha-2b in our patients originates from enhanced production by occult cancer cells, the sensitivity of the assay is augmented of approximately three times. Finally, it is possible that the response to rIFN-alpha-2b may be influenced by other factors such as the degree of differentiation of individual cancer, or intrinsic biological characteristics of the tumour as well as degradation rate of the 90K antigen.

The effect of rIFN-alpha-2b on 90K seems to have some specificity since neither CEA, nor CA 15-3, CA 19-9 or alpha-fetoprotein varied significantly during the 5 days of the test. A recent report (O'Connell *et al.*, 1989) has shown that the administration of rIFN-gamma to patients with advanced metastatic colorectal carcinoma can enhance the circulating levels of CA 19-9 and to a lesser extent of CA 15-3. Similar to our findings, the increase was seen within 24 h after drug administration but it was independent of basal antigen level. Further studies using different types of interferon as well as optimal doses and timing of administration are required.

In conclusion, this serum test with rIFN-alpha-2b may be indicative of disease recurrence in clinically NED cancer patients. Its value in this setting is the focus of a large prospective clinical trial now under way at the University of Chieti Medical School.

This work was supported by a grant of the Italian National Research Council (Progetto Finalizzato 'Applicazioni Cliniche della Ricerca Oncologica', IV sottoprogetto).

### References

ATTALLAH, A.M., NEEDY, C.F., NOGUCHI, P.D. & ELISBERG, B.L. (1979). Enhancement of carcinoembryonic antigen expression by interferon. *Int. J. Cancer*, **24,** 49–52.

BERGER, U., BETTELHEIM, R., MANSI, J.L., EASTON, D., COOMBER, R.C. & NEVILLE, A.M. (1988). The relationship between micrometastases in the bone marrow, histo-pathologic features of the primary tumor in breast cancer and prognosis. *Am. J. Clin. Path.*, **90,** 1–6.

BOYER, C.M., DAWSON, D.V., NEAL, S.E., WINCHEL, L.F., LESLIE, D.S., RING, D. & BAST, R.C. Jr (1989). Differential induction by interferons of major histocompatibility complex-encoded and non-major histocompatibility complex-encoded antigens in human breast and ovarian carcinoma cell lines. *Cancer Res.*, **49,** 2928–2934.

FISHER, E.R. & TURNBULL, R.B. Jr (1955). The cytologic demonstration and significance of tumor cells in the mesenteric venous blood of patients with colorectal carcinoma. *Sur. Gynecol. Obstet.*, **100**, 102–110.

GIACOMINI, P., IMBERT, L., AGUZZI, A., FISHER, P.B., TRINCHIERI, G. & FERRONE, S. (1985). Immunochemical analysis of the modulation of human melanoma-associated antigens by DNA recombinant immune interferon. *J. Immunol.*, **135**, 2887–2894.

GREINER, J.W., HAND, P.H., NOGUCHI, P., FISHER, P.B., PESTKA, S. & SCHLOM, J. (1984). Enhanced expression of surface tumor associated antigens on human breast and colon tumor cells after recombinant human leukocyte alpha-interferon treatment. *Cancer Res.*, **44**, 3208–3214.

GREINER, J.S., FISHER, P.B., PETSKA, S. & SCHLOM, J. (1986*a*). Different effects of recombinant human leukocyte interferons on cell surface antigen expression. *Cancer Res.*, **46**, 4984–4990.

GREINER, J.W., GUADAGNI, F., NOGUCHI, P., PETSKA, S., COLCHER, D., FISHER, B. & SCHLOM, J. (1986*b*). Recombinant interferon enhances monoclonal antibody targeting of carcinoma lesions *in vivo*. *Science*, **235**, 895–898.

GUADAGNI, F., SCHLOM, J., JOHONSTON, W.W. & GREINER, J.W. (1987). Recombinant human interferons mediate enhancement of tumor antigen expression on tumor cells isolated from pleural effusions and ascites. *J. Interferon Res.*, **7**, 798.

GUADAGNI, F., WITT, P.L., ROBBINS, P.F., SCHLOM, J. & GREINER, J.W. (1990). Regulation of carcinoembryonic antigen expression in different human colorectal tumor cells by interferon-gamma. *Cancer Res.*, **50**, 6248–6255.

GUESDON, J.L., TERNYCK, T. & AVRAMEAS, J. (1979). The use of avidin-biotin-interaction in immunoenzymatic techniques. *J. Histochem. Cytochem.*, **27**, 113–118.

IACOBELLI, S., ARNO', E., D'ORAZIO, A. & COLETTI, G. (1986). Detection of antigens recognized by a novel monoclonal antibody in tissue and serum from patients with breast cancer. *Cancer Res.*, **46**, 3005–3010.

IACOBELLI, S., SCAMBIA, G., NATOLI, C., BENEDETTI-PANICI, P., BAIOCCHI, G., PERRONE, L. & MANCUSO, S. (1988*a*). Recombinant human leukocyte interferon alpha 2b stimulates the synthesis and release of a 90K tumor-associated antigen in human breast cancer cells. *Int. J. Cancer*, **42**, 182–184.

IACOBELLI, S., ARNO', E., SISMONDI, P., NATOLI, C., GENTILONI, N., SCAMBIA, G., GIAI, M., CORTESE, P., BENEDETTI-PANICI, P. & MANCUSO, S. (1988*b*). Measurement of a breast cancer associated antigen detected by monoclonal antibody SP-2 in sera of cancer patients. *Breast Cancer Res. & Treat.*, **11**, 19–30.

LIAO, S.K., KWONG, P.C., KHOSRAVI, M. & DENT, P.B. (1982). Enhanced expression of melanoma-associated antigens and B2microglobulin on cultured human melanoma cells by interferon. *J. Natl Cancer Inst.*, **68**, 19–25.

MARTH, C., FUITH, L.C., BOCK, G., DAXENBLICHER, G. & DAPUNT, O. (1989). Modulation of ovarian carcinoma tumor marker CA-125 by gamma-interferon. *Cancer Res.*, **49**, 6538–6542.

MCCONAHEY, P.J. & DIXON, F.J. (1980). Radioiodination of proteins by the use of the chloramine-T method. In *Methods in Enzymology*, Vol. 70, Colowick, S.P. & Kaplan, N.O. (eds), pp. 210–213. New York: Academic Press.

O'CONNELL, M.J., RITTS Jr, R.A., SCHUTT, A.J., STEPHEN, A. & SHERWING, A. (1989). Recombinant interferon gamma lacks activity against metastatic colorectal cancer but increases serum levels of CA 19-9. *Cancer*, **63**, 1998–2004.

ROWLINSON, G., BALKWILL, F., SNOOK, D., HOOCKER, G. & EPENETOS, A.A. (1986). Enhancement by gamma interferon of *in vivo* tumor radiolocalization by a monoclonal antibody against HLA-DR antigen. *Cancer Res.*, **46**, 6413–6417.

SCAMBIA, G., BENEDETTI-PANICI, P., IACOBELLI, S., BAIOCCHI, G., BATTAGLIA, F., PERRONE, L., SONSINI, C., FERRANDINA, G., NATOLI, C. & MANCUSO, S. (1990). Recombinant alpha-2b-interferon enhances the circulating levels of a 90-kilodalton (K) tumor associated antigen in patients with gynecologic and breast malignancies. *Cancer*, **65**, 1325–1328.

SCAMBIA, G., BENEDETTI-PANICI, P., BAIOCCHI, G., GALLO, A., LAURELLI, G., IACOBELLI, S. & MANCUSO, S. (1991). Recombinant interferon alpha 2B dynamic test as a potential tool in the prediction of disease status at second-look in ovarian cancer. *Cancer*, **68**, 2582–2585.

SUTER, M., BUTLER, J.E. & PETERMAN, J.H. (1988). The immunochemistry of sandwich Elisa. III. The stoichiometry and efficacy of the protein-avidin biotin capture (PABC) system. *Mol. Immunol.*, **26**, 221–230.